# Effects of *Posidonia Oceanica* Beach-Cast on Germination, Growth and Nutrient Uptake of Coastal Dune Plants

**DOI:** 10.1371/journal.pone.0070607

**Published:** 2013-07-23

**Authors:** Silvia Del Vecchio, Núria Marbà, Alicia Acosta, Clara Vignolo, Anna Traveset

**Affiliations:** 1 Science Department, University of Rome 3, Rome, Italy; 2 Instituto Mediterráneo de Estudios Avanzados (CSIC-UIB), Esporles, Mallorca, Balearic Islands, Spain; Institute of Botany, Czech Academy of Sciences, Czech Republic

## Abstract

Seagrass meadows play an important role in marine ecosystems. A part of seagrass production is also exported to adjacent coastal terrestrial systems, possibly influencing their functioning. In this work we experimentally analyzed the effect of *Posidonia oceanica* beach-cast on plant germination, growth, and nutrient uptake of two plant species (*Cakile maritima* and *Elymus farctus*) that grow on upper beaches and fore dunes along the Mediterranean coasts. We compared plants growing in simple sand (control) with those growing in a substrate enriched with *P. oceanica* wrack (treatment) in laboratory. *P. oceanica* wrack doubled the N substrate pool and kept the substrate humid. Plants growing in the treated substrate grew faster, were twice as large as those growing in the control substrate, while tissues were enriched in N and P (*Cakile* by the 1.3 fold in N and 2.5 fold in P; *Elymus* by 1.5 fold in N and 2 fold in P). Our results suggest a positive effect of seagrass litter for the enhancing of dune species, highlighting its role for the conservation of coastal dune ecosystems.

## Introduction

Seagrasses grow along the coasts of all continents except Antarctica, where they form highly productive meadows [1] which carry out important coastal functions [2], [3]. They enhance coastal biodiversity by providing a habitat to numerous species and a nursery for a multitude of vertebrates and invertebrates [4], [5]. Seagrass meadows also stabilize the sea bed and protect the coastline from sea erosion [6]. In addition, they are hotspots for CO_2_ burial [7], [8] and also represent the basis of trophic chains for adjacent marine and terrestrial ecosystems [9], [10].

In the Mediterranean, the endemic species *Posidonia oceanica* (L.) Delile is the dominant seagrass, extending about 50.000 km^2^ [11]. *P. oceanica* ranks among the longest-living, forming millenary clones [12], and is one of the slowest-growing organisms in the Biosphere [12], [13]. This species is experiencing an overall decline throughout the Mediterranean basin due to local anthropogenic disturbances (e.g. [14], [15]) and climate change [16]. Because its ecological role in marine and terrestrial ecosystems and slow recovery time-scale, *P. oceanica* deserves special management and conservation attention [3], as the inclusion of *P. oceanica* beds in the EC Habitat Directive 92/43/EEC demonstrated [17].

Part of *P. oceanica's* biomass production is washed up on the beaches where it accumulates, forming deposits up to a few meters high. It is well known that seagrass beach cast provides refuge and food for coastal terrestrial fauna [18]–[21]. There is some evidence that seagrass wrack also influences coastal vegetation systems [22], although the specific effect on dune plant species remains unclear. Some authors support the hypothesis that *P. oceanica* wrack could be a source of N for coastal dune vegetation [22]. However, no studies have experimentally tested the specific response of beach and fore dune plants to substrate enrichment with *P. oceanica* wrack. By nutrient release and modifying the moisture of the substrate, seagrass wrack could influence plants during different stages of their life cycle. There is evidence that resource availability and drought constrain seed germination, seedling survival and establishment [23]–[25], leaf phenology (e.g. timing of leaf emergence), and plant growth rate and size [25]–[28]. Moreover, a fast - as opposed to slow - development during their early stages allows plants to acquire more resources and to increase their fitness [27], [29]. Affecting early stages of plant development, marine wrack might enhance the fitness and productivity of coastal dune plants. Hence, seagrasses may play a key role in systems beyond the marine environment such as terrestrial coastal ecosystems (beach and fore dunes, [3], [30]), particularly influencing plants during the early stage of their life-cycle.

*Cakile maritima* and *Elymus farctus* are among the most common species found in the upper beach (Habitat 1210) and the fore dune systems (Habitat 2110) in Mediterranean coastal areas [31], [32]. They usually grow close to the coastline on the upper beach and fore dunes, and due to wind transport receive greater amounts of seagrass wrack where *P. oceanica* meadows are present.

Here, we experimentally quantify the effect of *P. oceanica* wrack on seed germination and seedling survival, plant growth, nutrient acquisition and soil moisture on *Cakile maritima* Scop. and *Elymus farctus* (Viv.) Runemark ex Melderis to demonstrate that *P. oceanica* wrack contributes to enhance fitness and productivity of these target plant species. We do so by conducting a plant nursery experiment in order to avoid the influence of other environmental factors, and with seeds planted in a substrate (beach sand) enriched and not enriched with *P. oceanica* wrack.

## Methods

### Experimental design

In September 2009, we collected seeds of the annual *Cakile maritima* (hereafter referred as *Cakile*) and the perennial *Elymus farctus* (hereafter referred as *Elymus*) from the dune systems of Alcúdia Bay (north Mallorca, Balearic Islands, Western Mediterranean Sea). In the Mediterranean, *Cakile* is a typical upper beach species growing also on fore dunes while *Elymus* is a typical fore dune species [31], [33]. In April 2010, we collected sand and *P. oceanica* wrack deposited on the beach adjacent to the same dune systems. No specific permits were required for the described field studies. The field location where we collected the experimental plant material is not privately-owned, nor does it belong to a marine reserve. The study did not involve endangered or protected species. Although *P. oceanica* beds are included in the EC Habitat Directive 92/43/EEC, we only used seagrass debris that accumulates on beaches. In natural conditions, due to wave action seagrass debris accumulates on the lower part of the beach where vascular plants are usually absent. As a consequence of sun and wind exposure seagrass debris dry and are then transported by winds to the upper beach and fore dunes. To mimic these field conditions the collected *P. oceanica* debris were dried at 60°C for 48 h and measured for weight and volume. We prepared a mixed substrate of sand (previously homogenized) and *P. oceanica* wrack in the volumetric proportion of 1∶1. We estimated this proportion on the basis of field sampling. We collected sand and wrack in samples plots of 50 cm×50 cm, and 15 cm deep. The plots were located at different distances from the sea, from 10 m to 30 m, which corresponds to the distances at which the *Cakile* and *Elymus* communities are found in the study area. We measured the volume of sand and wrack in each sample. Then we calculated the mean volume of wrack and sand, and then the ratio between them. We observed that the proportion was about 1∶1, thus we considered this proportion for the treated (wrack-enriched) substrate in the plant nursery experiment. We used simple beach sand as the control substrate and the sand-wrack mix as the treated substrate. Then, 100 seeds of each selected species were sown in individual pots with control substrate and another 100 seeds of each species in the wrack-enriched substrate. We planted the seeds at a soil depth of 3 cm. Pots were all the same size (20 cm of diameter). In order to have the same substrate in each, both substrates (the control as well as the wrack-enriched substrate) were mixed up before filling in the pots. We watered the pots on alternate evenings, with fresh water (simulating rain) and using the same water quantity (150 ml). The experiment was carried out in an experimental plant nursery outside IMEDEA in Mallorca during spring and summer of 2010. Both species were planted simultaneously, on 4 May 2010.

### Germination and growth

We recorded the number of sprouted seedlings as well as the number of dead seedlings for each species. We measured the time elapsed since sowing until seedling appearance (i.e. “Germination delay”, in days) and the time elapsed until leaf appearance (“Timing of leaf emergence”, in days). Moreover, we measured plant height on each seedling on alternate days, to investigate changes in height over time.

For each species, we went on monitoring the plants until no germination events occurred for 10 days consecutively. Thus, we assumed that no more plants would germinate. Germination lasted 12 weeks for *Cakile* and 9 weeks for *Elymus*. As a consequence, we monitored each *Cakile* seedling for 12 weeks and each *Elymus* seedling for 9 weeks. In this way, we monitored the plants until they reached the same phenological stage: *Cakile* plants had fruits, while *Elymus* plants had no flowers. At the end of the monitoring period of each seedling and before removing the plants, we measured final plant height (H max) and total number of leaves. Due to the fast growth of *Cakile*, at the end of the monitoring period, we were also able to measure mean leaf area, total number of buds and total number of fruits in this species. Successively, we removed the plants, dried them at 60°C for 24 hours and measured their total biomass and nutrient concentration (see below).

Since *Cakile* germinated from 12 May to 25 July, we removed the plants from 4 August to 17 October. *Elymus* germinated from 19 May to 11 July, thus we removed the plants from 21 July to 12 September. The experiment lasted 24 weeks for *Cakile* and 19 weeks for *Elymus*, make it possible to assess germination and growth of *Elymus* and the entire life cycle (i.e. from germination to fructification) of *Cakile*. The experiment, therefore, was conducted during the major growing season of both species.

### Nutrient supply and water availability

We measured the concentration of N, C and P in ten dried samples of each species, and calculated the C/N ratio. To calculate the nutrient quantity provided by wrack, and to assess the differences from the control substrate, the nutrient concentration (N and P) and the C/N ratio of particulate material were also measured in control and wrack-enriched substrates before sowing (n = 10) as well as in *P. oceanica* wrack. Powdered samples were analyzed for C and N content using a CHN analyzer (Fisons NA1500). P content was determined by a dry-oxidation, acid hydrolysis extraction followed by a colorimetric analysis of phosphate concentration of the extract [35].

In order to determine whether *P. oceanica* wrack provided the N acquired by the experimental plants, as suggested by previous studies [22], we measured the isotopic abundance of ^15^N in 10 selected plants harvested at the end of the experiment, as well as in control and wrack-enriched substrates and *P. oceanica* wrack at the beginning. We analyzed the isotope abundance of ^15^N in dried ground sample material using a standard elemental analyzer isotope ratio mass spectrometer (EA-IRMS) procedure, as described by Fourqurean et al. [36]. The EA was used to combust the organic material and to reduce the formed gases into N_2_, which were measured on a Finnigan MAT Delta C IRMS in a continuous flow mode. Stable isotope abundances were expressed in the delta notation (δ^15^N), according to the equation 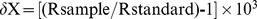, where R is the corresponding ratio ^15^N/^14^N and R_standard_ was atmospheric N (air, N_2_).

Additionally, we monitored substrate moisture with a sensor (Theta Probe, type ML2x) in order to investigate if wrack in the substrate influences plant water availability. We measured substrate moisture daily, in the morning, during one entire week, in 18 control and 18 treated pots (9 for each species) in the plant nursery.

### Data analysis

In the case of germination, we first verified if *P. oceanica* wrack influenced germination percentage and seedling survival by means of Chi-square tests [37].

For each species, we calculated Absolute Growth Rate in height (AGR_H_, cm day-1) and Absolute Growth Rate in biomass (AGR_B_, g day-1) for the entire growth period as,
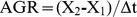
where X_2_ is either height or dry weight, respectively, of the plant at the end of the observation period and X_1_ is that at the beginning of the observation period. Relative Growth Rate (RGR_H_, day-1) at weekly intervals was defined as [34],
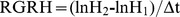



In order to highlight differences between control and treatment in germination delay, growth parameters (H max, timing of leaf emergence, total leaves, biomass, AGR_H_, AGR_B_ and RGR_H_ for each week, leaf area, total buds, total fruits) and nutrient content (N, P, C/N) as well as ^15^N abundance in the plants and in substrate treatments, we adopted a null model approach. When we compared independent samples, all response variables proved to be non-normally distributed (assessed by Shapiro-tests and visual estimation, data not shown). We used a Monte Carlo *F*-test for two groups by performing matrix permutations, running separate analysis for each species [38]. We contrasted the observed *F* value with those simulated by 3×10^4^ random permutations. This number of permutations avoids algorithm biases [39]. *F* index was calculated for the original data as well as for the simulated matrix; results were then compared, calculating the probability (*P*) of the null hypothesis that the observed *F* index (*F_obs_*) was drawn at random from the distribution of the simulated *F* indexes (*F_exp_*). Non-random differences were assumed when P _Fobs ≥ Fexp_ ≤0.05 [40]. We applied the Bonferroni correction for multiple comparisons in interpreting the *P* values to reduce type I errors [37].

Furthermore, for each species we performed a linear mixed effect model (LME) to investigate whether changes in height over time were significantly different between control and treated plants. We used weekly values of the plant height as response variables, the presence of wrack (control vs treatment) and the time after germination (week number) as fixed factors. Individual plant identity was used as a random factor in the model in order to account for repeated measures on the same plant over time. If there is an effect of wrack on plant height over time, we expect to observe a significant interaction term of wrack × time.

Since substrate moisture levels can depend not only on the presence of wrack but also on the climatic conditions of the day, a General Linear Model (GLM) was used to determine the effects of both wrack and the day of measurement on substrate moisture in the pots.

All the statistical analyses were performed using Statistica for Windows (version 7.0), except the analyses of growth, nutrient and isotopes concentration data, which were performed using Ecosim 7.0 [38] and the LME, which was performed in the R statistical framework (“nlme” package).

## Results

### Influence on seed germination and seedlings survival

The presence of *P. oceanica* wrack did not influence final germination percentage and survival of seeds of either of the two species (χ^2^ test: *P*>0.01). On average, seed germination was 16% in *Cakile* and 41% in *Elymus* (Table 1). However, wrack delayed germination time in both species (by two-fold in *Cakile* and three-fold in *Elymus*).

**Table 1:** Results of Monte Carlo *F*-test for two groups on growth parameters in *Cakile and Elymus*.

	Cakile maritima				Elymus farctus			
	Control	Treatment			Control	Treatment		
Germination (%)	17	15			45	37		
	Observed Mean		F-obs	F-exp	Observed Mean		F-obs	F-exp
Germination delay (days)	18.5	54.4	75.63**	1.06	35.1	59.8	57.18*	1.02
H max (cm)	30.73	61.3	13.07**	1.04	19.66	40.04	97.05*	1.04
Timing of leaf emergence (days)	15.7	8.6	5.02	1.07	12.17	10.52	3.87	1.01
Total leaves	9.94	22.77	29.33**	1.04	2.71	3.71	33.98*	1.01
Biomass (g)	0.22	0.53	12.15**	1.04	0.05	0.11	44.63*	1.01
AGR_B_ (g day^−1^)	0.003	0.009	58.3**	1.1	0.001	0.002	44.63*	1.01
AGR_H_ (cm day^−1^)	0.34	0.68	54.85**	1.08	0.31	0.63	97.05*	1.04
RGR_H_ (day^−1^)								
Week 1	0.17	0.15	0.6	1.06	0.22	0.24	1.37	1.03
Week 2	0.04	0.07	11.08	1.08	0.06	0.05	0.54	1.01
Week 3	0.01	0.05	20.93**	1.08	0.04	0.08	8.89*	1.01
Week 4	0.04	0.04	6.07	1.08	0.025	0.024	0.17	1.02
Week 5	0.04	0.07	3.91	1.05	0.02	0.02	0.03	1.02
Week 6	0.06	0.05	1.01	1.06	0.01	0.01	0.07	1.03
Week 7	0.07	0.03	7.08	1.07	0.02	0.02	0.93	1.03
Week 8	0.05	0.02	15.7**	1.07	0.02	0.01	0.15	1.01
Week 9	0.03	0.008	5.50	1.06	0.006	0.007	0.45	1.05
Week 10	0.01	0.003	4.09	1.06	–	–	–	–
Week 11	0.007	0.003	2.28	1.13	–	–	–	–
Week 12	0.005	0.002	1.29	1.02	–	–	–	–
Leaf area (cm^2^)	1.4	4.99	75.53**	1.08	–	–	–	–
Total buds	18.05	56.46	38.94**	1.08	–	–	–	–
Total fruit	5.86	12.84	21.87**	1.06	–	–	–	–

** = *P*<0.002;

* = *P*<0.003.

The critical *P*-value of 0.05 was corrected using a Bonferroni correction yielding a *α* value of 0.002 in the case of *Cakile* and a *α* value of 0.003 in the case of *Elymus*.

### Effects on plant growth

Conversely to the effect on final germination, the presence of *P. oceanica* wrack did exert a significant positive effect on the growth parameters of both species (Table 1). Plants of *Cakile* and *Elymus* growing in wrack-enriched substrate were larger (in height and mass), developed a higher number of total leaves and exhibited higher AGR_H_ and AGR_B_ at the end of the experiment than did those growing in the control substrate. In both species, the fastest RGR_H_ was observed during the third week of seedling development. In *Cakile*, seedlings growing in the control substrate grew faster than those growing in the wrack-enriched substrate during the eighth week. Individuals of this species growing in wrack-enriched substrate achieved a larger leaf area and a higher number of total buds and fruits than those growing in control substrate (Table 1).

In both *Cakile* and *Elymus* species, treated plants showed significant higher growth trajectories during the entire observation period (LME model; *Cakile*: wrack × time t-value_358_  = 14.2; *P*<0.001. *Elymus*: wrack × time t-value_635_  = 20.3; *P*<0.001).

### Nutrient and moist conditions in experimental substrates

**Figure 1:**
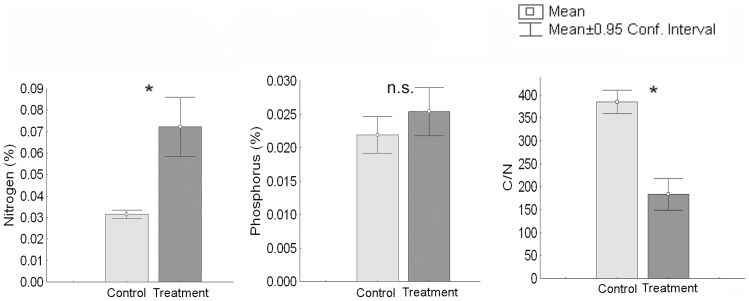
Substrate nutrient concentration before sowing. Values are mean ±0.95 confidence interval (n = 5). The critical *P*-value of 0.05 was corrected using a Bonferroni correction yielding a *α* value of 0.01. *  = *P*<0.01; n.s., not significant.

*Posidonia oceanica* wrack contained 0.66% (of dry weight) of N and 0.04% of P, and had a d^15^N of 4.37 ‰. The addition of wrack to the substrate enhanced N substrate pool (Figure 1). At the beginning of the experiment, N concentration in *P. oceanica* wrack-enriched substrate was two-fold higher than in control substrate (Figure 1). Accordingly, C/N ratio was lower in wrack-enriched than in control substrate (Figure 1). Conversely, substrate enrichment with *P. oceanica* wrack did not enhance substrate phosphorous concentration (Figure 1). At the end of the experiment, N concentration decreased by 25% in treated substrate (Monte Carlo *F*-test for two groups; *Cakile*: pseudo-F = 13.3, *P* = 0.01; *Elymus*: pseudo-F = 10.8, *P* = 0.01; data not shown), whereas in control substrate it was not different from that before sowing in any of the experimental species (Monte Carlo *F*-test for two groups; *Cakile*: pseudo-F = 0.04, *P* = 0.9; *Elymus*: pseudo-F = 3.2, *P* = 0.2). During the experiment, similar phosphorous substrate concentrations were maintained across species and treatments (Monte Carlo *F*-test for two groups; *P*>0.1 in any case; data not shown).

**Figure 2:**
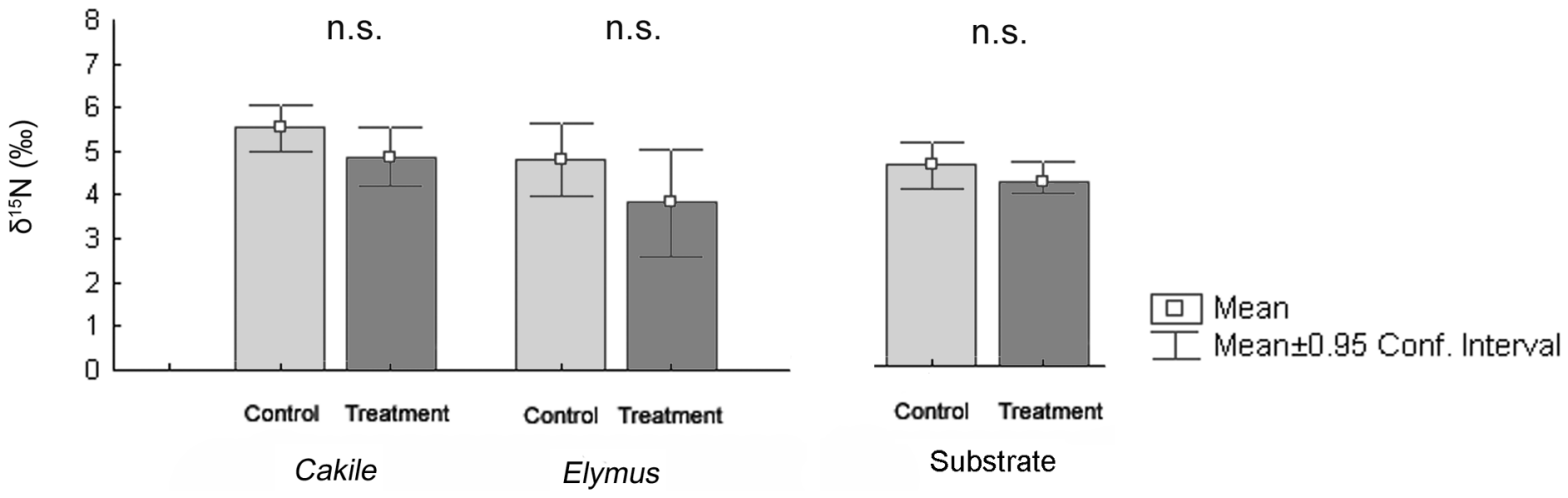
Stable isotopes abundance in the dried plants and in the substrate before sowing. Values are mean ±0.95 confidence interval (n = 10); n.s., not significant.

In particulate organic matter in wrack-enriched and control substrate the natural abundance of ^15^N was similar to that of *P. oceanica* wrack (Figure 2), demonstrating that *P. oceanica* wrack was the N source in both experimental substrates.

Moisture was significantly influenced by the presence of *P. oceanica* wrack (F_1,238_  = 152.64; *P*<0.0001) with higher values in the treated pots. Even though these values depended on the day of measurement (F_6,238_  = 16.17; *P*<0.0001), the effect of wrack was much stronger.

### Contribution to plant nutrient uptake

**Figure 3:**
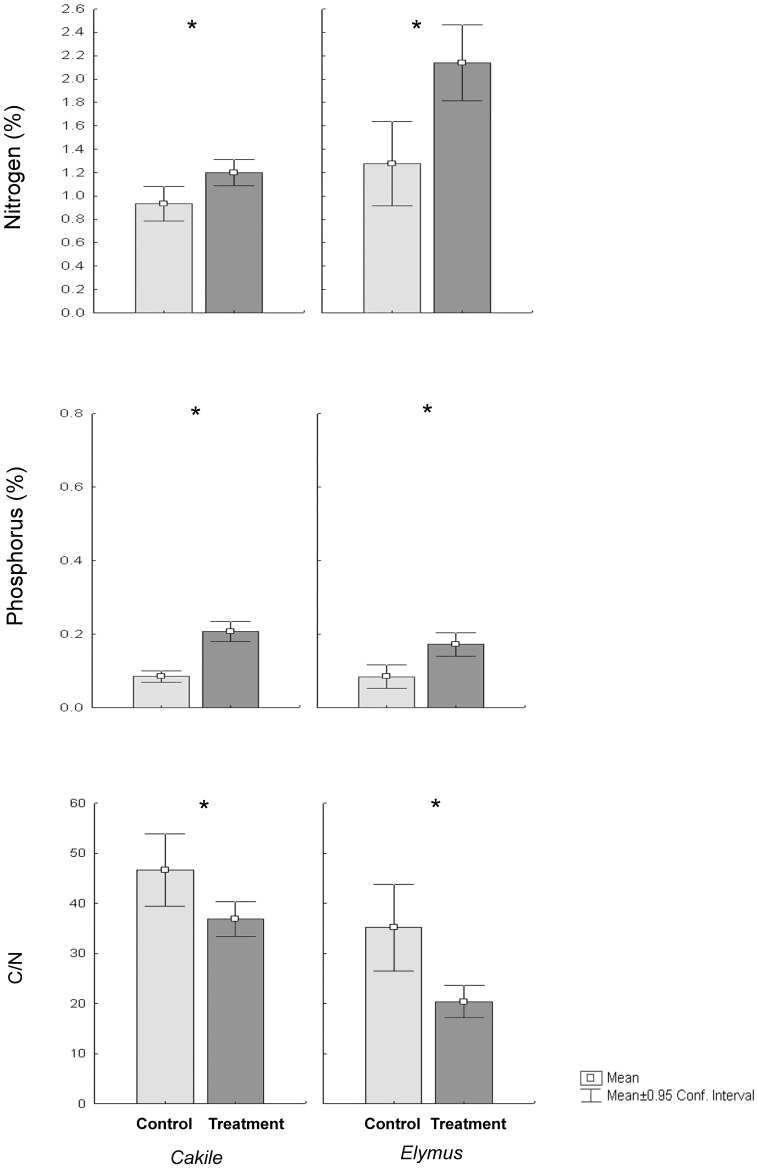
Plant nutrient concentration for each of the two study species for control and treated plants. Values are mean ±0.95 confidence interval (n = 10) The critical *P*-value of 0.05 was corrected using a Bonferroni correction yielding a *α* value of 0.01. * *P*<0.01; n.s., not significant.

The tissues of treated plants were N enriched when compared to those of control plants, since N concentration in plants of *Cakile* and *Elymus* growing on wrack-enriched substrate were 1.3 and 1.5 fold higher than those on control plants (Figure 3). As a consequence, plants growing in wrack-enriched substrate had a lower C/N ratio than those growing on control substrate (Figure 3). There were no differences in isotope abundance of ^15^N in the plants growing in *P. oceanica* wrack enriched and control substrates (Figure 2). Plants of both species growing in wrack-enriched substrate had twice the amount of P found in plants growing in substrate control (Figure 3).

## Discussion

### Influence of wrack on seed germination and seedlings survival

We found that *P. oceanica* wrack is related to the germination delay in both studied species even though the wrack exerted no influence on the final germination percentage. The seagrass wrack accumulated on the seashore is sea-water soaked, thus salty [10]. A possible explanation of the germination delay may be due to the high concentration of NaCl supplied by *P. oceanica* wrack to the substrate. Different authors have previously found that salt induces seed dormancy in psammophilous species [41], [42]. In fact, this dormancy induction has been considered to be a mechanism to guarantee a higher seedling survival rate. Fresh water provided by rainfall leaches out the salt and allows germination, preventing seedlings from emerging during the dry and warm summers, characteristic of the Mediterranean climate [43], [44]. Thus, our findings suggest that beach-cast *P. oceanica* material is a further control factor for the germination delay in beach and fore dune vegetation.

### Influence of wrack on plant growth

Growth of the two species was significantly enhanced by the presence of *P. oceanica* wrack. On the whole, plants growing in wrack-enriched substrate grew faster and were bigger than those growing in control substrate. Despite the different growth rates and sizes of *Cakile* and *Elymus* species, plant size of both species responded similarly to *P. oceanica* wrack enrichment, developing individuals twice as large as those in control substrate.

*P. oceanica* wrack increased RGR_H_ during the third week of seedling growth in both species, endowing the plants with a better growth performance. Fast-growing plants, in fact, appear to gain more access to resources, are able to overcome substrate water deficiency and, consequently, begin a positive feedback mechanism of seedling establishment [27], [28]. In particular, *Cakile* plants growing in wrack-enriched substrate underwent a rapid growth earlier than the control ones (week 3 *vs* week 8). As soon as seedlings achieve their fastest growth, plants are able to develop larger leaves and a higher number of flowers and fruits during their life span, so guaranteeing them a higher reproductive capacity [29].

### The contribution of wrack to nutrient and water supply

Our results support the hypothesis that wrack influences dune species through the direct provision of nutrients, while also helping to prevent substrate water loss.

Wrack-enriched substrate had higher N content and a lower C/N ratio compared to simple sand (control). In the wrack-enriched substrate, besides a higher nutrient availability, it is also possible that plant nutrient uptake was facilitated according to the different substrate conditions. It is well known that plant nutrient uptake depends on the substrate microbial activity levels: the higher the activity levels, the faster is the organic matter decomposition, and hence the more rapid the nutrients availability [45]–[48]. Since a greater microbial activity occurs when the C/N ratio in the substrate is low and the beach cast wrack is metabolically very active [10], [45], [48], it is likely that the microbial activity was higher in the treated substrate compared to the control, promoting in this way a higher nutrient uptake. A high microbial activity in the substrate can reduce the substrate pH values, through the release of intense CO_2_ fluxes [10]. Consequently, the substrate of coastal dunes, which is typically alkaline, could have reached neutral pH values through the addition of organic matter [49]–[51]. The substrate pH is a further factor that controls nutrient assimilation; in particular, P uptake is known to be favored in neutral conditions [52]. Thus, in the wrack-enriched substrate, it is likely that plant nutrient uptake was enhanced by both a higher microbial activity and a decay in substrate pH. This hypothesis, however, needs to be experimentally confirmed in future studies.

The higher N content in *Cakile* and *Elymus* probably stimulated plant growth as N availability controls the rate of photosynthesis [53]. In fact, in a previous study on *Cakile* and *Salsola kali,* the addition of N in the substrate increased concentration of N in the plant tissues' photosynthetic ability [53]. In the treated plants of both species we found a lower C/N ratio, suggesting that treated plant incorporated more N in relation to C. A low C/N ratio in the plant tissues indicates that plants are not N-depleted [54]. Our results suggest that control plants suffered N deficiency if compared to treated plants: this further supports the hypothesis that in nutrient poor habitats, such as sandy coasts, *P. oceanica* wrack could be an important source of N for the vegetation of both beach and fore dune.

We observed no increment in P in treated substrate, although we did find significant higher concentration of P in *Cakile* and *Elymus* which grew in the wrack-enriched substrate. P is a limiting element in coastal dune systems [55]; because P concentration in seagrass leaves is almost one order of magnitude lower than that of N [56], P release was much lower than that of N. However, the P enrichment of treated plant tissues suggests that in the absence of *P. oceanica* wrack, availability of this nutrient might limit dune plant growth. *P. oceanica* wrack, therefore, also enhanced P availability or at least indirectly favored its assimilation, probably due to an increment of microbial activity and a pH decrement in the wrack-enriched substrate.

The findings that ^15^N abundance in plants growing in wrack-enriched and in control substrates was similar reveals that seagrass litter was the main source of N acquired by *Cakile* and *Elymus*. Despite the control substrate not being experimentally enriched with *P. oceanica* wrack, its isotopic N abundance at the beginning of the experiment was similar to that of wrack-enriched substrate as well as that of the *P. oceanica* wrack itself. This finding confirms that, even at low concentration, the particulate N present in the control substrate originated from *P. oceanica* debris.

The presence of wrack in the substrate can ameliorate physical stress by increasing the moisture [57]; our results further suggest that besides nutrients, treated plants seem to benefit from indirect higher water availability. Thus, *P. oceanica* influences dune vegetation not only by providing it with nutrients but also by preventing substrate aridity.

### Final considerations and conservation perspectives

Coasts represent the boundary where marine and terrestrial environments meet. These environments are linked through the transfer of energy and nutrients [58]. Sandy beaches are dynamic harsh environments, nutrient poor and exposed to extreme environmental conditions [10], [59], [60]. Our results show that the accumulation of wrack on beaches influence not only terrestrial animal species [5], [61], [62], but also the primary production of beach and fore dune vegetation.

Our results provide strong evidence that the presence of *P. oceanica* wrack enhances plant growth of species growing in the upper beach as well as in the fore dune; the wrack fertilizes the substrate and keeps it moist, while also favoring plants nutrient uptake. In addition, we can now hypothesize that the presence of wrack on beaches, because of its function in enhancing sand humidity, helps to reduce soil aridity, a phenomenon expected to increase due to climate changes in the Mediterranean [63]. These positive effects of *P. oceanica* wrack on species growth should be taken into account when planning conservation measures for dune ecosystems.

Many coastal areas depend on the income derived from recreational use of beaches, which attract many visitors especially during the summer months [64], [65]. Our results demonstrate that the ecological functioning of the beaches and adjacent dunes may also depend on the seagrass beach cast deposits, these unfortunately being often removed to increase the beaches' appeal to holyday-makers [66]. In light of seagrass wrack's influence on coastal ecosystems and of the benefits on coastal plants, such beach-cast wrack removal represents a further negative impact on these highly already endangered ecosystems, further hindering these ecosystems' long term preservation.
